# Dolutegravir is not associated with weight gain in antiretroviral therapy experienced geriatric patients living with HIV

**DOI:** 10.1097/QAD.0000000000002853

**Published:** 2021-02-23

**Authors:** Giovanni Guaraldi, Stefano Calza, Jovana Milic, Andrea Calcagno, Emanuele Focà, Matteo Rota, Stefano Renzetti, Anna Celotti, Matteo Siano, Benedetto Maurizio Celesia, Stefania Piconi, Giuseppe Vittorio de Socio, Anna Maria Cattelan, Giancarlo Orofino, Agostino Riva, Silvia Nozza, Giovanni di Perri

**Affiliations:** a Department of Surgical, Medical, Dental and Morphological Sciences, University of Modena and Reggio Emilia, Modena; b Department of Molecular and Translational Medicine, University of Brescia, Brescia; c Unit of Infectious Diseases, Department of Medical Sciences, University of Turin, Turin; d Division of Infectious and Tropical Diseases, Department of Clinical and Experimental Sciences, University of Brescia and ASST Spedali Civili of Brescia, Brescia; e Third Division of Infectious Diseases, University of Milan, Ospedale L. Sacco, Milan; f ARNAS ’Garibaldi’ UOC Malattie Infettive Catania, Catania; g First Division of Infectious Diseases Unit, University of Milan, Ospedale L. Sacco, Milan; h Department of Medicine, Infectious Diseases Unit, Azienda Ospedaliera di Perugia and University of Perugia, Santa Maria Hospital, Perugia; i Unit of Infectious Diseases, Department of Internal Medicine, Azienda Ospedaliera-Universitaria di Padova, Padova; j Unit of Infectious Diseases, Division A, Ospedale Amedeo di Savoia, Turin; k Department of Infectious Diseases, San Raffaele Scientific Institute, Milan, Italy.

**Keywords:** >65, dolutegravir, geriatric, integrase strand transfer inhibitor, weight gain

## Abstract

The aim of this study was to explore weight gain in people with HIV (PWH) at least 65 years of age who switch to a DTG based regimen (DTG-s) vs. remaining INSTI-naive (INSTI-n) on stable ART.

**Methods:**

This was a longitudinal prospective study of PWH from the GEPPO cohort. At the beginning of the observational period, participants were INSTI-naives (INSTI-n). During follow-up, they were divided in two groups: INSTI-n vs. dolutegravir-switchers (DTG-s) with no further change in ART. Body weight was assessed at baseline and at last follow-up visit. Significant weight gain was defined as an increase at least 5% of baseline weight from the first to the last visit. ART regimens were collected at each patients’ visit. Kaplan--Meier curves were drawn to assess time to reach a weight gain more than 5%.

**Results:**

Out of 568 PWH (83.1% men, median age 69.5 years), 427 (75%) were INSTI-n and 141 (25%) DTG-s. After an average follow-up of 2.6 (±0.8) years, no significant change in body weight was observed both among INSTI-n [delta weight = 0.02 (±7.5), *P* = 0.633] and DTG-s [delta weight = −0.04 (±5.2), *P* = 0.755]. Weight gain was also not significantly different between study groups (9.3% in INSTI-n and 15.1% in DTG-S: *P* = 0.175). No significant differences in time to achieve a weight gain greater or equal than 5% of baseline weight emerged in INSTI-n vs. DTG-s (*P* = 0.93), two-drug regimens (2DR) vs. three-drug regimens (3DR) (*P* = 0.56) or TAF vs. TDF (*P* = 0.56).

**Conclusion:**

Results from a large Italian cohort did not show a significant weight gain associated with switch to DTG in PWH 65 years of age or older. This finding emerged also when comparing 3DR vs. 2DR and TAF exposed and unexposed geriatric PWH.

## Background

The obesity epidemic is a major public health problem affecting both industrialized and developing countries, exposing individuals to a higher risk of atherosclerosis, cancer, neurodegeneration and type II diabetes, that in turn also relates to ageing and contributes to lessening life expectancy [1].

Recent studies postulated that obesity is a mirror of ageing and that the mechanisms by which the comorbidities of obesity and ageing develop are similar [2]. Results from the InCHIANTI Study, a longitudinal Italian study has shown that an increase in BMI is associated with an increase in epigenetic age acceleration [3].

Changes in body composition, including obesity, with ageing have been widely described in people with HIV (PWH) [4,5]. The level of complexity of this interaction is further increased due to a possible association between modern antiretroviral therapy (ART) initiation, or switching, and weight gain.

In an AIDS Clinical Trial Group (ACTG) study of ART initiation in resource-diverse settings (A5175), more than 25% of participants were classified as overweight or obese at study entry, and approximately 40% of participants were overweight or obese by week 144 [6]. In the ADVANCE study, an open-label phase III randomized trial conducted in South Africa, patients randomized to dolutegravir (DTG)-based regimens, especially in combination with tenofovir alafenamide (TAF), experienced a significantly higher weight gain as compared to those randomized to tenofovir disoproxil fumarate (TDF)-based regimens and to the standard-of-care. This weight gain was greatest among female patients, that is 6.4 and 3.2 kg at week 48 for DTG combined to TAF as compared to TDF, respectively [7]. However, some other reports show that the rate of weight gain is not higher in PWH switching to integrase strand transfer inhibitor (INSTI) [8].

The progressive increase in body weight raises concern regarding the possibility of an obesity epidemic affecting PWH, as well as its possible association with accelerated ageing and other comorbidities [4]. However, it is still unclear whether weight gain observed in PWH treated with INSTIs is associated with higher incidence of diabetes, nonalcoholic fatty liver disease or other metabolic conditions [9].

No randomized clinical studies assessed weight gain in geriatric PWH on ART. Data can be obtained from observational studies. One of the largest geriatric HIV cohorts is the Geriatric Patients Living with HIV/AIDS (GEPPO) cohort, an Italian prospective multicentre cohort started in 2015, recruiting HIV-positive geriatric PWH aged 65 years or older and HIV negative controls [10,11].

This study aims to explore weight gain in PWH aged at least 65 years who switch to a DTG-based regimen (DTG-s) in comparison with PWH who remained INSTI-naive (INSTI-n) on stable ART.

## Materials and methods

This was a longitudinal prospective multicentral study of PWH from the Geriatric Patients Living with HIV/AIDS (GEPPO) cohort. Characteristics of this cohort are described elsewhere [12] and comprises ART-experienced men and women PWH older than 65 years, regardless of viro-immunological status.

### Inclusion and exclusion criteria

At the beginning of the observational period (baseline), participants were INSTI-naives (INSTI-n). During follow-up, they were divided into two groups: INSTI-n vs. dolutegravir-switchers (DTG-s) with no further change in ART. Inclusion criteria was availability of at least two body weight measure. Body weight was assessed at baseline and at last follow-up visit. In the DTG-s group, the baseline visit was prior to switch.

### Outcome measure

Excess weight gain was defined as an increase of more or equal than 5% body weight from the first (baseline) to the last visit (end of follow up). ART regimens were collected at each visit by keeping track of the date of start of any drug. Regimens were classified as two-drug regimen (2DR) if included either one INSTI or one protease inhibitor and one nonnucleoside reverse transcriptase inhibitor (NNRTI) or lamivudine (3TC). Regimens were classified as three-drug regimen (3DR) if they included one INSTI and two nucleoside reverse transcriptase inhibitors (NRTI), or one protease inhibitor boosted with ritonavir or cobicistat and two NRTI or one NNRTI and two nucleoside reverse transcriptase inhibitors (NRTI). Current exposure to TDF, TAF or abacavir (ABC) was specified.

### Covariates

Demographic, anthropometric and HIV-related variables were collected at baseline and during follow-up. These included age, sex, BMI and smoking status. HIV variables included current and nadir CD4^+^ T-cell counts, CD4/CD8 ratio, plasma HIV RNA viral load. The duration of HIV infection was calculated as the time between diagnosis and the last visit.

Noninfectious comorbidities (NICM) was defined according to EACS guidelines [13]. Cardiovascular disease (CVD) included myocardial infarction, coronary artery disease, peripheral vascular disease, stroke and angina pectoris, as well as coronary artery bypass grafting and angioplasty, based on diagnoses recorded in patient charts. Hypertension was defined as two consecutive measurements of blood pressure more than 140/90 mmHg; dyslipidaemia as elevated total or low-density lipoprotein (LDL) cholesterol or low high-density lipoprotein (HDL) cholesterol using ATPIII classification; type 2 diabetes mellitus (T2DM) as fasting serum glucose levels more than 126 mg/dl or HbA1C more than 6.5%; chronic kidney disease (CKD) as an estimated glomerular filtration rate (eGFR) of less than 60 ml/min/1.73 m^2^ calculated using the CKD-Epi equation; osteoporosis in postmenopausal women and men aged at least 50 years as a bone mass index (BMD) *T*-score of −2.5 or less, and in premenopausal women and men aged less than 50 years as a BMD *Z*-score of −2 or less in addition to a fragility fracture; chronic obstructive pulmonary disease (COPD) as postbronchodilator spirometric forced expiratory volume in the first second/forced vital capacity (FEV1/FVC) less than 0.70. Liver cirrhosis was defined through noninvasive assessment with either serum biomarkers (FIB-4) [14] or transient elastography, and cancers including both AIDS and non-AIDS defining conditions. Multimorbidity was defined as presence at least three comorbidities in the same individual. Polypharmacy was defined as at least five drug components other than ART.

### Statistical analyses

Results were expressed as mean and standard deviation (±SD) for continuous variables and as frequencies for categorical variables. Comparisons of demographic, anthropometric and HIV characteristics were done through the nonparametric Mann--Whitney test for continuous variables and the χ^2^ test for categorical variables. The McNemar test for categorical and the Wilcoxon signed-rank test for continuous variables were used for outcome comparison between last follow-up and baseline visit separately within INSTI-n and DTG-s patients. Kaplan--Meier curves were drawn to assess time to reach a weight gain more than 5%, and curve comparison across strata defined by drug regimens (INSTI-n vs. DTG-s, TAF vs. TDF or 2DR vs. 3DR) were based on the log-rank test. A *P* value less than 0.05 was deemed to be statistically significant.

## Results

The GEPPO cohort included 568 geriatric PWH (83.1% men) followed for a mean period of 2.6 (±0.8) years, 427 (75%) of whom were in the INSTI-n group and 141 (25%) were in the DTG-s group. The average number of weight measurements 2.75 (range 2–4).

Supplementary Figure 1 presents the most common 2DR and 3DR regimens in both groups.

Cox proportional hazard model adjusted for sex, age, multimorbidity, weight at the baseline and time between baseline visit and DTG initiation did not find overall difference between study groups, although in univariate analyses prevalence of CKD, osteoporosis, hypertension, cancer and multimorbidity wherein higher in DTG-s group (Supplementary table 1).

No differences in demographic, immunovirological and polypharmacy prevalence were observed between the two groups (Table 1).

**Table 1:** Demographic, anthropometric and HIV characteristics of geriatrics people living with HIV in the GEPPO cohort both at baseline and follow-up.

	INSTI-n (*N* = 427)	DTG-s (*N* = 141)	*P*
Age (years), mean (SD)	73.3 (4.7)	73.5 (4.7)	0.65
Males (%)	360 (84.3%)	112 (79.4%)	0.19
HIV exposure (years), mean (SD)	18.9 (7.1)	18.8 (7.4)	0.78
Body weight at first visit (kg), mean (SD)	74.5 (13.9)	71.8 (13.4)	0.09
HIV undetectability at first visit, %	95%	92.9%	0.99
Current CD4^+^ cell count at first visit (cells/μl), mean (SD)	650.7 (235.4)	652.9 (277)	0.69
Nadir CD4^+^ cell count at first visit (cells/μl), mean (SD)	246.94 (185.87)	204.17 (159.87)	0.05
Any virologic failure, *N* (%)	1 (0.2%)	0 (0.0%)	0.99
ART regimen at baseline visit			
PI, %	191 (44.7%)	77 (54.6%)	0.04
NNRTI, %	215 (50.4%)	49 (34.8%)	< 0.001
Current TDF exposure, %	148 (34.7%)	44 (31.2%)	0.47
Current ABC exposure, %	129 (30.2%)	53 (37.6%)	0.12
Comorbidities, baseline visit			
Chronic kidney disease, %	90 (27.8%)	56 (44.1%)	0.001
Osteoporosis, %	74 (23.8%)	44 (35.5%)	0.02
Cardiovascular disease, %	36 (11.4%)	13 (10.3%)	0.87
Dyslipidaemia, %	162 (52.6%)	73 (61.3%)	0.11
Lipodystrophy, %	103 (35.2%)	42 (37.5%)	0.73
Type 2 diabetes mellitus, %	81 (25.3%)	25 (20.2%)	0.25
Hypertension, %	208 (63.6%)	93 (73.8%)	0.05
Cancer, %	46 (14.4%)	35 (27.1%)	0.004
Cirrhosis, %	26 (8.0%)	5 (3.9%)	0.15
Chronic obstructive pulmonary disease, %	17 (5.4%)	6 (4.8%)	0.85
Multimorbidity (at least three comorbidities), %	165 (38.6%)	79 (56.0%)	< 0.001
Polypharmacy (>five medications), %	81 (19.0%)	30 (21.3%)	0.612
Metabolic variables at first visit			
Total cholesterol (mg/dl), mean (SD)	173.53 (156.76)	156.00 (72.67)	0.89
LDL cholesterol (mg/dl), mean (SD)	112.85 (32.74)	128.18 (32.24)	0.15
HDL cholesterol (mg/dl), mean (SD)	50.08 (12.43)	51.26 (14.60)	0.73
Body weight at last visit			
Body weight (kg), mean (SD)	74.1 (14.9)	71.0 (13.3)	0.06
Delta weight (kg), mean (SD)	0.31 (5.8)	−0.04 (5.2)	0.97
Weight gain >5% between first and last visit, %	18 (8.8%)	13 (15.1%)	0.14
HIV undetectability at last visit, %	92.6%	96.0%	0.44
Follow-up (years), mean (SD)	2.53 (0.83)	2.78 (0.89)	0.001
ART regimen at last visit			
PI, %	133 (31.1%)	8 (5.7%)	<0.001
NNRTI, %	216 (50.6%)	13 (9.2%)	< 0.001
Current TAF exposure, %	141 (33.0%)	36 (25.5%)	0.12
Current TDF exposure, %	38 (8.9%)	0 (0.0%)	< 0.001
Current ABC exposure, %	103 (24.1%)	43 (30.5%)	0.13
Metabolic variables at last visit			
Total cholesterol (mg/dl), mean (SD)	142.94 (101.65)	125.78 (59.58)	0.13
LDL cholesterol (mg/dl), mean (SD)	108.66 (34.89)	105.95 (33.54)	0.36
HDL cholesterol (mg/dl), mean (SD)	51.38 (14.86)	48.91 (14.95)	0.13

The prevalence of comorbidities is provided only for baseline visit. HDL, high-density lipoprotein; LDL, low-density lipoprotein.

The average follow-up was 2.6 years, while the range was 4 years, with 3 months longer duration in DTG-s group. Mean weight at baseline was 74.5 (±13.9) kg in INSTI-n and 71.8 (±13.4) kg in DTG-s (*P* = 0.09) and no difference was observed.

There were no significant differences in body weight at last follow-up as compared to the baseline body weight both in INSTI-n [delta weight = 0.02 (±7.5), *P* = 0.633] and DTG-s patients [delta weight = −0.04 (±5.2), *P* = 0.755].

Weight gain (≥5%) was not significantly different between INSTI-n and DTG-s patients (*P* = 0.175) neither were the prevalence of obesity from first to last visit in each group (McNemar test *P* = 0.773 and *P* = 1 for INSTI-n and DTG-s, respectively) nor between them (*P* = 0.997 at last visit) (Supplementary figure 2). At last follow-up visit, there was no significant difference in CD4 (673 vs. 663, *P* = 0.8) nor virologic suppression (96.3% vs. 96.2%, *P* = 0.99).

**Fig. 1:**
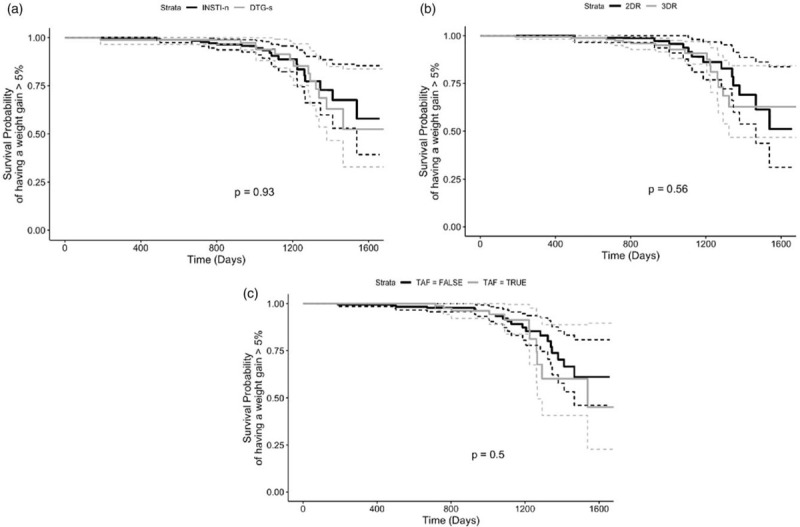
Kaplan--Meier plots showing time to reach a weight gain at least 5% across INSTI-n vs. DTG-s (a), TAF vs. TDF (b) or 2DR vs. 3DR (c).

The time-to-event analysis did not show a significant difference in time to achieve a weight gain greater or equal than 5% of baseline weight among INSTI-n vs. DTG-s (*P* = 0.93), 2DR vs. 3DR (*P* = 0.56) or TAF vs. TDF (*P* = 0.56) (Fig. 1).

Exploratory analyses from the same dataset using the third quartile as cut-off for the weight change or the continuous variable of body weight gain as outcomes confirmed the same results.

## Discussion

Our analysis using data from the GEPPO cohort did not support a significant weight gain associated with switch to DTG in PWH over 65 years of age. This topic is very popular in the recent HIV literature [15,16], although we are still seeking for a reliable weight gain definition and we are still exploring the clinical implications of this condition.

If, as presumed, weight gain is associated with cardiometabolic conditions and premature ageing, clinical relevance may be higher in older adults with HIV experiencing higher burden of comorbidities and accentuated ageing. To explore the impact of DTG, we use as comparison PWH who remained INSTI-n. However, our study did not reveal any association between use of DTG and weight gain in PWH more than 65 years.

We observed that DTG-s PWH at baseline had higher burden of multimorbidity and lower nadir CD4^+^ cell count, possibly identifying a more vulnerable population with a greater immunological ‘scar’ and potentially higher risk of cardiometabolic alterations. DTG offered to these individuals the unique opportunity to avoid toxicities related to TDF-based or ABC-based regimens and also justifies the higher proportion of 2DR regimens observed in this study arm.

Bictegravir, also belonging to INSTI class, has been investigated in a small, phase 3b, multicentre, open-label, single arm study [17]. Among the 86 PWH older than 65 years switching from E/C/F/TAF or FTC/TDF and third agent to BIC/FTC/TAF, no weight change was observed at 24 and 48 weeks of follow-up [17].

Our real-life data from a larger geriatric cohort show similar results for DTG. The absence of a consistent number of individuals who reached weight gain did not allow us to explore the relative contribution of multiple risk factors. Nevertheless, a time-to-event analysis did not reveal a significant difference in time to achieve a weight gain greater or equal than 5% of baseline weight comparing not only the two study arms but also among two strategic ART regimens, 2DR vs. 3DR and TAF-based vs. TAF-spearing regimens.

Recently, the AGEhIV cohort, which included 598 HIV-positive and 550 HIV-negative individuals aged more than 45 years, analysed 119 HIV-positive participants who switched to an INSTI-containing regimen (53% dolutegravir; 35% elvitegravir; 13% raltegravir). A bodyweight increase greater than 5% occurred in 28 out of 119 (23.5%) PWH after INSTI initiation as compared to 31 out of 238 (13%, *P* = 0.013) nonswitching PWH, and in 28 out of 238 (11.8%, *P* = 0.005) HIV-negative controls, suggesting that clinically relevant weight gain upon switching to INSTI may be a relatively rare phenomenon deserving further investigation [18]. Given that the pathogenetic mechanism leading to weight gain in PWH is still unclear, we cannot identify the role of geriatric specific protective factors that prevent this phenomenon.

This study has several limitations including a low prevalence of women included in the cohort and outcome definition. Weight gain defined as a 5% increase in body weight is opposed to the benefit of 5% weight loss obtained with lifestyles interventions in cardiometabolic conditions. Secondly, weight is not an ideal anthropometric measure; nevertheless, it is still used in large epidemiological studies to calculate and categorize BMI to define obesity. Moreover, in the geriatric and as well as in the oncological setting, the lack of weight gain does not automatically exclude fat accumulation while losing lean mass [19].

In the future, we plan to include body composition data acquired with DEXA in the GEPPO cohort and analyse relationship between lower BMI and risk of mortality, as suggested in the general population [20]. This tool will allow us to verify a stable lean and fat mass in geriatric patients switching to DTG and exclude a fat mass increase parallel to sarcopenia.

The implication of these results in clinical practice stands on the fact that DTG is one of the most prescribed INSTI agents both in industrialized and developing countries. Evidence from GEPPO cohort suggest that weight gain should not be considered as a side effect of DTG in geriatric PWH.

## Acknowledgements

We thank PENTA Foundation for the GEPPO cohort support.

Other members of the GEPPO Study Group: Francesco Castelli, Anna Carla Chiesa from the Division of Infectious and Tropical Diseases, Department of Clinical and Experimental Sciences, University of Brescia, Italy; Micol Ferrara, Stefano Bonora from the Unit of Infectious Diseases, Department of Medical Sciences, University of Turin, Italy; Antonella Castagna, Andrea Poli, Nadia Galizzi from the Department of Infectious Diseases, San Raffaele Scientific Institute, Milan, Italy; Marinello Serena from Unit of Infectious Diseases, Department of Internal Medicine, Azienda Ospedaliera-Universitaria di Padova, Italy; Marco Nofri, Daniela Francisci, Marta Baroni from Azienda Ospedialiera and University of Perugia, Italy; Andrea Marino, Bruno Cacopardo from the Division of Infectious Diseases, University of Catania, ARNAS Garibaldi, Catania, Italy; Gervasi Elena, Massimo Galli from the First Division of Infectious Diseases Unit, University of Milan, Ospedale L. Sacco, Milan, Italy; Chiara Mussi from the Orthogeriatric Unit, Department of Mother, Child and Adult Medicine and Surgical Science, University of Modena and Reggio Emilia, Italy.

This study was not sponsored. This study was carried out as part of the authors’ routine work.

### Conflicts of interest

G.G. received research grant and speaker honorarium from Gilead, ViiV, MERCK and Jansen. He attended advisory boards of Gilead, ViiV and MERCK. AC received research grants from Gilead and ViiV and speaker's honoraria from Gilead, Insmed, Janssen-Cilag, MSD and ViiV. E.F. received research grants from Gilead and ViiV, speaker's honoraria from Gilead, Janssen-Cilag, Menarini, MSD and ViiV and attendee advisory boards of Gilead, Janssen-Cilag and Viiv. G.V.D.S. attended advisory board of Janssen-Cilag and ViiV. S.N. received research grant from Gilead, speaker's honoraria Gilead, Janssen-Cilag, MSD and ViiV.

## Supplementary Material

Supplemental Digital Content

## Supplementary Material

Supplemental Digital Content

## Supplementary Material

Supplemental Digital Content
