# Supplementary material for: Dolutegravir is not associated with weight gain in antiretroviral therapy experienced geriatric patients living with HIV
Source: AIDS. 2021 Feb 23;35(6):939–45. doi: 10.1097/QAD.0000000000002853 (PMC9904432; doi:10.1097/QAD.0000000000002853)

**Supplementary figure 1** shows proportion of 2DR and 3DR regimens in INSTI-n PLWH (panel A) and proportion of 2DR and 3DR regimens in DTG-s (panel B).


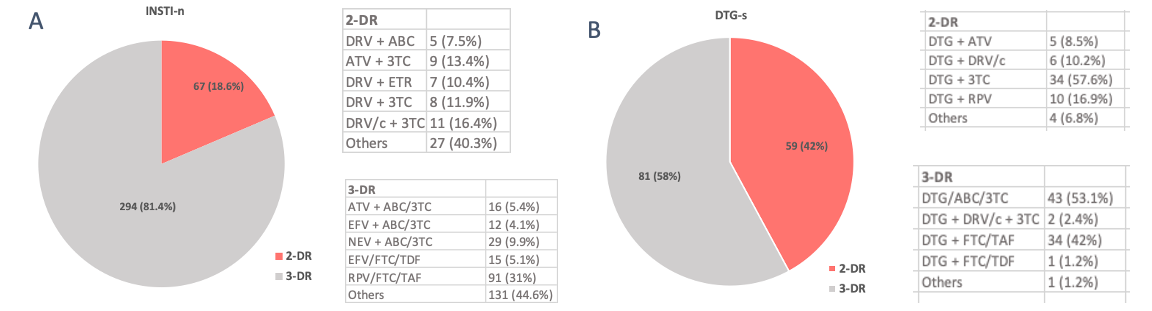

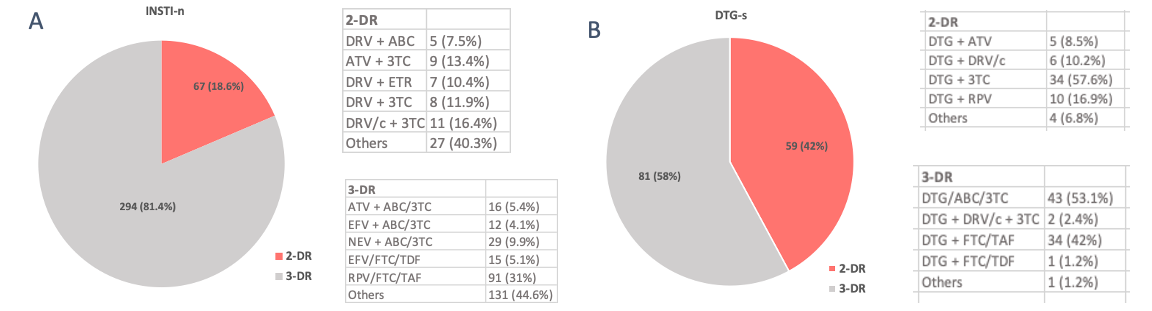

Supplement: Supplemental Digital Content [file aids-35-939-s001.doc]
