# Supplementary material for: Dolutegravir is not associated with weight gain in antiretroviral therapy experienced geriatric patients living with HIV
Source: AIDS. 2021 Feb 23;35(6):939–45. doi: 10.1097/QAD.0000000000002853 (PMC9904432; doi:10.1097/QAD.0000000000002853)
Supplement: Supplemental Digital Content [file aids-35-939-s002.doc]

**Supplementary table 1.** Cox proportional hazard model adjusted for sex, age, multimorbidity, weight at the baseline and time between baseline visit and DTG initiation.

| *Predictors* | *Estimates* | *CI* | *p* |
| --- | --- | --- | --- |
| DTG-s vs INSTI-n | 1.47 | 0.47 – 4.55 | 0.509 |
| Males vs Females | 2.33 | 0.69 – 7.90 | 0.174 |
| Age | 1.04 | 0.96 – 1.13 | 0.334 |
| Multimorbidity | 0.84 | 0.40 – 1.79 | 0.659 |
| First visit weight | 0.96 | 0.93 – 0.99 | **0.019** |
| Time before DTG initiation | 0.87 | 0.54 – 1.40 | 0.558 |
