# Supplementary material for: Dolutegravir is not associated with weight gain in antiretroviral therapy experienced geriatric patients living with HIV
Source: AIDS. 2021 Feb 23;35(6):939–45. doi: 10.1097/QAD.0000000000002853 (PMC9904432; doi:10.1097/QAD.0000000000002853)

**Supplementary figure 2** depicts categories of BMI at the first and the last visit in both study arms. Panel A shows both study arms at first visit. Panel B shows both study arms at last visit. Panel C shows INSTI-n group at both baseline and follow-up. Panel D shows DTG-s at both baseline and follow-up.


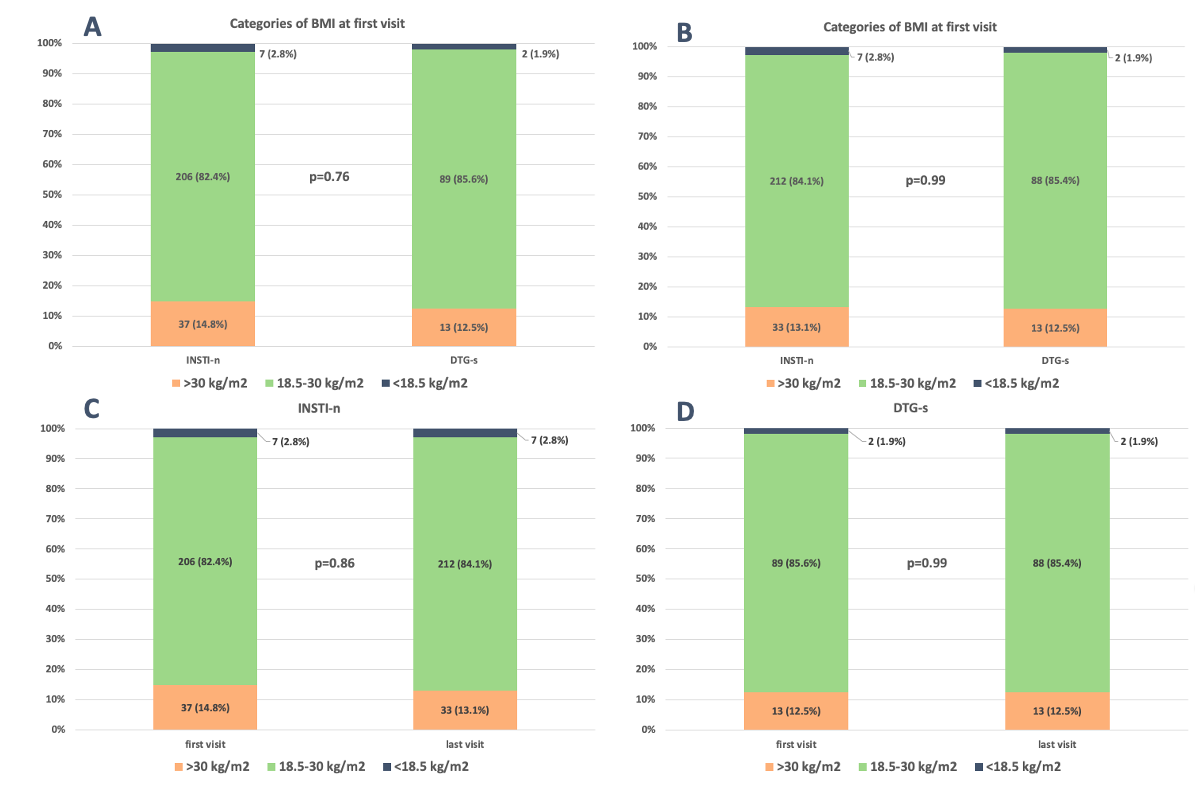

Supplement: Supplemental Digital Content [file aids-35-939-s003.doc]
